# Uncontrolled Asthma Among Children and Its Association With Parents’ Asthma Knowledge and Other Socioeconomic and Environmental Factors

**DOI:** 10.7759/cureus.35240

**Published:** 2023-02-20

**Authors:** Ahlam Mazi, Fatema Madani, Ebtehag Alsulami, Abeer Almutari, Rawan Alamri, Jana Jahhaf, Samahir Alsulaimani

**Affiliations:** 1 Department of Pediatrics, King Abdulaziz University Faculty of Medicine, Jeddah, SAU; 2 Department of Pediatric Medicine, Al Aziziyah Children Hospital, Jeddah, SAU; 3 Department of Family Medicine, King Abdulaziz University Faculty of Medicine, Jeddah, SAU; 4 Department of Anesthesia, King Faisal Specialist Hospital and Research Centre, Jeddah, SAU; 5 Department of Internal Medicine, King Abdulaziz University Faculty of Medicine, Jeddah, SAU

**Keywords:** saudi arabia, use of oral steroids, exposure to bakhoor, asthma control medication, breathing distress, respiratory system, parental knowledge, uncontrolled asthma, children

## Abstract

Objective

To estimate the frequency of uncontrolled asthma among asthmatic children from Jeddah and to analyze its association with parental asthma knowledge and other socioeconomic and environmental factors.

Method

A cross-sectional study was conducted at the Pediatrics Departments of King Abdulaziz University Hospital (KAUH) in Jeddah, Saudi Arabia, from July to December 2018. It involved the caregivers of 150 children with asthma, who were following at KAUH. A structured questionnaire was administered by a phone interview to collect the following: socioeconomic and environmental factors of asthma, answers to the Arabic version of the Asthma Control Test^TM^ (ACT), and answers to the Arabic version of the caregiver Asthma Knowledge questionnaire (AKq).

Result

The frequency of uncontrolled asthma was 32.7% (95%CI: 25.2 - 40.8). Parents had myths about asthma such as “children with asthma should use asthma control medications (inhaled corticosteroids) only when they have symptoms” and “it’s not good for children to use the inhaler for too long”. Besides, we observed mixed results regarding parents’ knowledge about the disease, with correct answers ranging from 56.0% to 88.7% depending on the item. Exposure to bakhoor (aromatic woodchips) at home (OR = 0.41, p=0.044), two or more ICU admissions during the past 12 months (OR = 3.30, p=0.030), and using a rescue inhaler even if there’s no cough or wheeze when the child gets the flu (OR = 0.22, p=0.001) were the three independent factors of uncontrolled asthma among children.

Conclusion

Uncontrolled asthma concerns one-third of the asthmatic children following at our centre, representing a less concerning figure compared to the national data. The contribution of parents’ knowledge to asthma control did not show significant results, although uncontrolled asthma may represent an opportunity to increase parents’ knowledge and awareness. We emphasize the significance of exposure to bakhoor, the use of oral steroids, and the number of ICU admission as strong indicators for uncontrolled asthma in children. An adaptive national strategy should be designed to enable effective and personalized interventions, resources, and objectives for maximized benefits.

## Introduction

Asthma is one of the most common chronic diseases in the world and in Saudi Arabia, and represents a significant public health concern [1,2]. The Saudi Initiative for Asthma Updated Report 2021 (SINA-2021) indicated that the local prevalence of asthma is increasing, with children being a highly vulnerable group [3]. Studies carried out over the past three decades showed that the overall prevalence of asthma in the pediatric population ranged between 8% to 25% with an increasing trend [4-14]. Classically, asthma is described as a chronic hypersensitivity-induced airway inflammation that leads to mucus hypersecretion, smooth muscle hypertrophy, and subepithelial fibrosis, which in turn causes airway lumen narrowing [15].

According to the Global Initiative for Asthma (GINA) guidelines of 2021, children aged 6-11 years with asthma should be given inhaled corticosteroids (ICS) in association with short-acting β2-agonist (SABA) either as maintenance or reliever therapy (i.e., for mild asthma) [16]. In children aged ⩽5 years, it is recommended to use inhaled SABA alone for managing wheezing episodes; whereas in case of overt asthma, and/or frequent or severe respiratory/wheezing episodes maintenance therapy can be indicated for a limited duration of three months. Due to these new recommendations, the terms “intermittent” and “mild persistent” asthma are no longer used in the GINA classification considering that the previous classification did not consider a differential response to ICS [16].

Achieving adequate long-term control is the main desired outcome of asthma management. Immunohistochemical analysis of bronchial and transbronchial biopsies found an increased deposition of myofibroblasts and extracellular matrix molecules including versican, decorin, biglycan, and collagen in the central and distal lung of patients with uncontrolled asthma, compared with those who had controlled asthma [17]. This suggests a greater pulmonary tissue remodelling and, therefore, a higher likelihood of irreversible loss of lung elasticity and function. In children, poorly controlled asthma exposes them to multiple risks such as reduced cardiovascular performance, obesity, learning disabilities, as well as impaired growth [18,19]. On the other hand, the course, severity, and controllability of asthma in children are determined by a plethora of socioeconomic and environmental factors [20-22].

A study from Saudi Arabia revealed that the number of siblings and adequate asthma knowledge in caregivers improved the quality of asthma control in asthmatic children aged 4-11 years. The same study showed that a household income of less than Saudi Riyal (SAR) 15,000 and sharing a bedroom with a sibling increased the risk of uncontrolled asthma [22]. These data stress the involvement of the family environment in influencing the odds of uncontrolled asthma. Additionally, parental level of awareness and attitudes toward childhood asthma is a paramount determinant of uncontrolled asthma among afflicted children.

In a study from China, parents caring for 2960 asthmatic children aged 1-14 years responded to a questionnaire that tested their knowledge and attitudes about asthma management. Remarkably, the authors found that the levels of parental knowledge and attitudes were positively associated with the results of pulmonary function tests, in addition to other indicators of asthma control such as regular physicians’ visits, proper self-monitoring (through the peak flow meter and the Children’s Asthma Control Test questionnaire), avoidance of asthma triggers (such as smoking), use of inhaled β2 receptor agonist, and adherence to prescribed medication [23]. On the other hand, a cross-sectional study from Riyadh, Saudi Arabia, demonstrated that inappropriate knowledge among caregivers (of them, 97.5% were mothers) about the adverse effects of long-term therapy in asthmatic patients increased the prevalence of uncontrolled asthma by three-fold [24]. Therefore, identifying the gaps in knowledge and misbeliefs about asthma among parents of asthmatic children as well as the environmental factors of uncontrolled asthma is crucial to improve asthma control and mitigate the long-term burden of the disease.

The present study estimated the frequency of uncontrolled asthma among asthmatic children from Jeddah, Saudi Arabia, and analyzed its association with parental knowledge about asthma and other environmental factors. 

## Materials and methods

Design and setting

The study was approved by the Institutional Review Board of the Faculty of Medicine, King Abdulaziz University Hospital (KAUH), Jeddah, Saudi Arabia, and conformed to the ethical standards of the Helsinki Declaration. It was a descriptive and analytical, cross-sectional study conducted at the Pediatrics Departments of KAUH from July to December 2018.

Population and sampling

The study involved the caregivers of all children with asthma aged 3-18 years who were following at KAUH pediatric clinics. The number of eligible participants was 150, which was the targeted sample size. Asthma was diagnosed at the hospital according to the Saudi Initiative for Asthma Group (SINA) guidelines 2016 [3].

Data collection

A structured questionnaire was administered through a phone interview with all participants. The questionnaire comprised five sections.

(i) Section one collected socioeconomic factors including the child’s age, gender, nationality, city, father’s and mother’s age and education level, household income, and the number of siblings.

(ii) Section two explored environmental factors of asthma including smoking and bakhoor (aromatic woodchips) use at home, the presence of plants at home, living near an industrial zone, exposure to farm animal or pets including cats during the first year of life, and home hygiene (dust, mold, carpet, etc.).

(iii) Section three explored asthma-specific and nonspecific medical history including age at onset, asthma duration, oral corticosteroids use in the past 12 months, antibiotics use in the past 12 months, number of intensive care unit (ICU) admissions in the past 12 months, history of allergic rhinitis, atopic dermatitis, gastroesophageal reflux disease (GRD), and breastfeeding duration.

(iv) Section four consisted of the Asthma Control Test^TM^ (ACT) using both the 4-11 years and 12 years and older versions as applicable. Both versions enabled the calculation of a score and a score of 19 or less was indicative of uncontrolled asthma. The questionnaire is validated and has shown high diagnostic accuracy in discriminating controlled from uncontrolled asthma [25]. The Arabic version of the ACT was used in the present study [26].

(v) Section five consisted of the Arabic version of the Asthma Knowledge Questionnaire (AKq) for use with parents or guardians of children with asthma to measure the knowledge of caregivers [27]. The scale comprises 17 Likert-type scale items, with graded answers from 1 (strongly disagree) to 5 (strongly agree). The items were divided into three dimensions including myths and beliefs (seven items), knowledge about asthma (six items), and asthma-related notions (four items). A scoring system was used to dichotomize the participants’ answers into correct or incorrect depending on the item. For positive items 8-13 and 16, an answer of ‘‘strongly agree’’ or ‘‘agree’’ was considered correct. For negative items including 1-7, 14-15, and 17, an answer of ‘‘strongly disagree’’ or ‘‘disagree’’ was considered correct. In either case, “neutral” answers were considered incorrect.

Procedure

Caregivers were contacted by the investigators and informed consent was obtained verbally prior to the administration of the questionnaire. The participants were given enough time to respond to each question and were informed of their right to withdraw from the survey at any time without this compromising the management of their child. Data was collected directly on a preformatted electronic form.

Statistical analysis

Data were analyzed using IBM SPSS Statistics for Windows, Version 21.0 (Released 2012; IBM Corp., Armonk, New York, United States). Descriptive statistics were carried out to present the answers to the different sections of the survey. The internal consistency of both ACT and AKq was analyzed by calculating Cronbach’s alpha. Depending on their ACT score, participants were dichotomized into uncontrolled (ACT score ≤19) and controlled asthma (ACT score >19). The two groups were compared for the different child- and family-related and environmental factors using independent t-test for continuous variables and chi-square test for categorical variables. Since the internal consistency of AKq was low (Cronbach's alpha<0.70), denoting low intercorrelation between the scale item, an overall asthma knowledge score could not be calculated. Thus, the association of parental knowledge with the level of asthma control was analyzed for every knowledge item separately using chi-square test. A multivariate logistic regression model, including the significant factors in bivariate analyses, was used to analyze the independent factors associated with uncontrolled asthma. The statistical significance was set for a p-value <0.05.

## Results

Socioeconomic and environmental data

Parents of 150 asthmatic children completed the survey. The children’s mean age was 8.71 years (standard deviation (SD)=3.91), and 98 (65.3%) of them were male. The majority were born in Jeddah city (82.7%). The mean (SD) father’s and mother’s ages were 42.84 (8.28) years and 36.52 (6.47) years, respectively. Parents’ education showed a high percentage of education among fathers (40.1%) and mothers (38.6%), and 47.3% reported a low household income (<5000 SAR per month). The majority of the children had three or more siblings (64.7%) (Table 1).

**Table 1:** Sociodemographic and environmental parameters n: frequency; %: the percentage; SAR: Saudi Riyal

Factor	Categories	
Child-related factors		
Age (years), mean (SD)		8.71 (3.91)
Gender, n (%)	Boy	98 (65.3)
	Girl	52 (34.7)
Nationality, n (%)	Saudi	94 (62.7)
	Non-Saudi	56 (37.3)
City of birth, n (%)	Jeddah	124 (82.7)
	Other city	22 (14.7)
	Abroad	4 (2.7)
Family-related factors		
Father’s age (years), mean (SD)		42.84 (8.28)
Father’s education, n (%)	Primary education	7 (4.7)
	Secondary education	83 (55.3)
	Diploma	16 (10.7)
	Bachelor's degree	37 (24.7)
	Master's or PhD degree	7 (4.7)
Mother’s age (years), mean (SD)		36.52 (6.47)
Mother’s education, n (%)	Primary education	12 (8.0)
	Secondary education	80 (53.3)
	Diploma	9 (6.0)
	Bachelor's degree	47 (31.3)
	Master's or PhD degree	2 (1.3)
Household income (SAR), n (%)	Low <5000	71 (47.3)
	Average 5000 - 10000	54 (36.0)
	High > 10000	25 (16.7)
Number of Siblings, n (%)	None	7 (4.7)
	1-2	46 (30.7)
	3-4	60 (40.0)
	5+	37 (24.7)
Environmental factors		
Parental smoking, n (%)	Yes	51 (34.0)
	No	99 (66.0)
Other family member smoking, n (%)	Yes	61 (40.7)
	No	89 (59.3)
Presence of plants at home, n (%)	Yes	11 (7.3)
	No	139 (92.7)
Living near industrial zone, n (%)	Yes	15 (10.0)
	No	135 (90.0)
Exposure to pets or farm animals, n (%)	Yes	12 (8.0)
	No	138 (92.0)
Cat at home during the first year of life, n (%)	Yes	11 (7.3)
	No	139 (92.7)
Cat at home in the last 12 months, n (%)	Yes	5 (3.3)
	No	145 (96.7)
Bakhoor at home, n (%)	Yes	104 (69.3)
	No	46 (30.7)
Home hygiene (dust, mold, carpets, etc.), n (%)	Yes	51 (34.0)
	No	99 (66.0)

Regarding environmental factors, we observed the frequency of parental smoking (34.0%) and other family member smoking (40.7%); besides the in-house exposure to bakhoor (69.3%), plants (7.3%), cats (3.3%), and other pets or farm animals (8.0%). Additionally, 7.3% of the parents reported the presence of a cat at home during the first years of the child’s life (Table 1).

Asthma-specific and other health-related parameters

The mean (SD) age at asthma onset was 2.23 (2.14) years, resulting in a mean disease duration of 6.26 years on the day of the survey. During the past 12 months, 29.3% and 55.3% of the children have used oral corticosteroids and antibiotics, respectively, and 30.7% have been admitted to the intensive care unit (ICU) at least once. Asthma-specific and other health-related parameters are presented in Table 2.

**Table 2:** Asthma-related and other health-related parameters (N=150) n: frequency; %: percentage § diabetes mellitus, hypertension, hyperlipidemia, rheumatological disorder, cardiac, etc.

Factor	Categories	
Asthma-specific parameters		
Age at asthma onset (years), mean (SD)		2.23 (2.14)
Asthma duration, mean (SD)		6.26 (3.54)
Oral corticosteroid use in the past 12 months, n (%)	No	106 (70.7)
	Yes	44 (29.3)
Duration	Daily (continued)	5 (3.3)
	≤ 1 month	6 (4.0)
	>1-3 months	13 (8.7)
	>3 months	15 (10.0)
	Not documented	5 (3.3)
Antibiotic use in the past 12 months, n (%)	Yes	83 (55.3)
	No	67 (44.7)
Number of ICU admissions in the past 12 months, n (%)	None	104 (69.3)
	1	19 (12.7)
	2+	27 (18.0)
Other health parameters, n (%)		
History of allergic rhinitis	Yes	58 (38.7)
	No	92 (61.3)
History of atopic dermatitis	Yes	25 (16.7)
	No	125 (83.3)
Gastroesophageal reflux disease	Yes	16 (10.7)
	No	134 (89.3)
Other chronic diseases^ §^	Yes	11 (7.3)
	No	139 (92.7)
Breastfeeding	Yes	110 (73.3)
	No	40 (26.7)
Breastfeeding duration	Not applicable	40 (26.7)
	< 3 months	23 (15.3)
	3-6 months	20 (13.3)
	6-12 months	27 (18.0)
	> 12 months	40 (26.7)

 Parents’ knowledge about asthma

**Figure 1:**
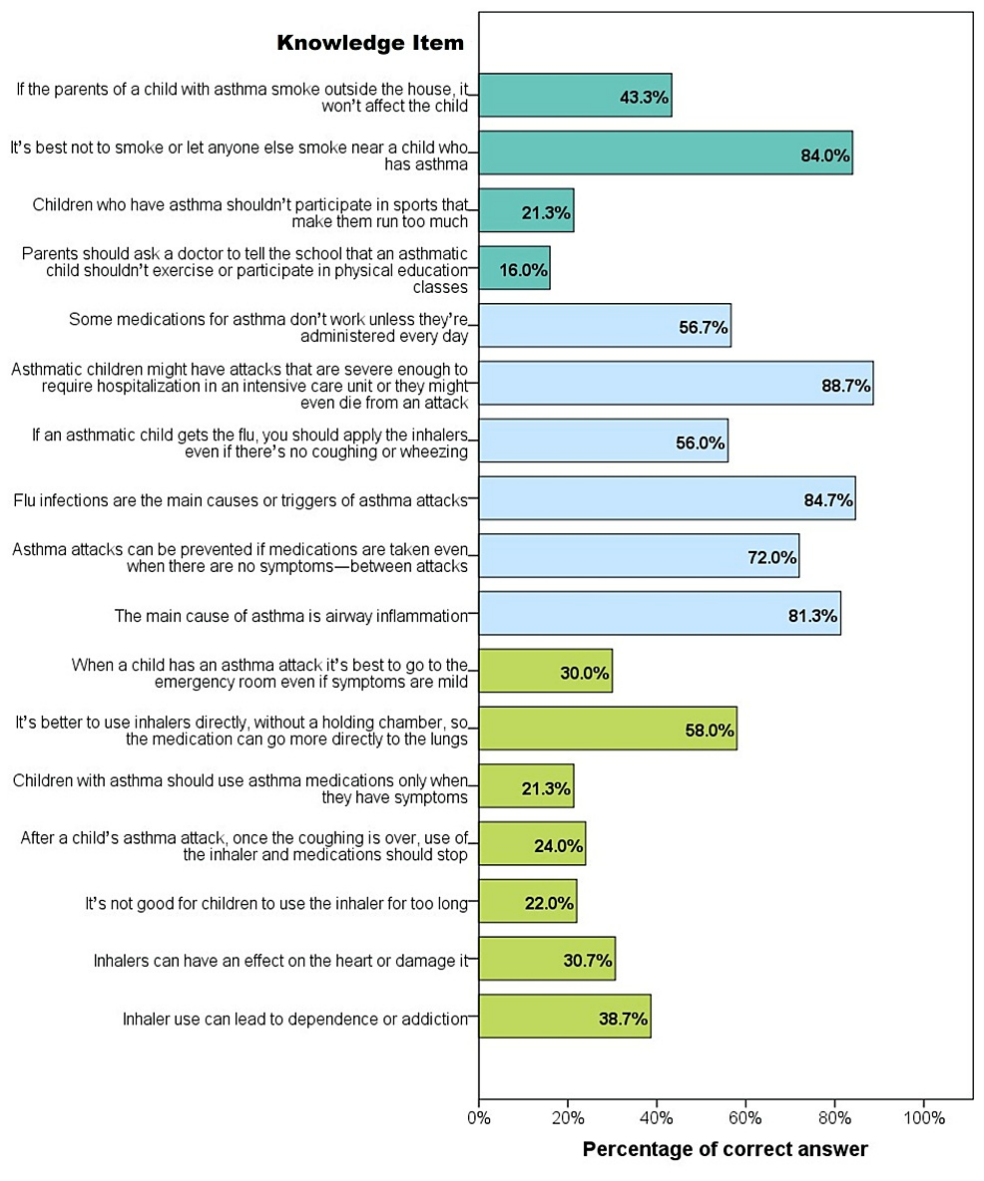
Knowledge about asthma among parents of afflicted children Bars represent the percentage of participants who provided a correct answer to the given knowledge item. Knowledge items are divided into three subscales: Myths and beliefs regarding asthma (light green bars); knowledge about the disease (light blue bars); and knowledge about the associated aspects of asthma (dark green bars).

The percentages of correct answers to the AKq items are shown in Figure 1. In the myths & beliefs subscale, the items with the lowest correctness rates were “children with asthma should use asthma medications only when they have symptoms”, “it’s not good for children to use the inhaler for too long”, and “after a child’s asthma attack, once the coughing is over, use of the inhaler and medications should stop” (21.3%, 22.0%, and 24.0%, respectively).

Regarding the knowledge subscale, the percentages of correct answers ranged from 56.0% for “if an asthmatic child gets the flu, you should apply the inhalers even if there’s no coughing or wheezing” (the correct answer is yes), to 88.7% for “asthmatic children might have attacks that are severe enough to require hospitalization in an ICU or they might even die from an attack” (the correct answer is yes).

Regarding the knowledge about the associated aspects subscale, the majority (84.0%) of the participants admitted that smoking near the asthmatic child was not recommended while 43.3% believed smoking outside the home would not affect the child. Furthermore, only a minority correctly rejected the absolute prohibition of sports among asthma children and that parents should ask a doctor to prohibit the child’s participation in physical education classes (21.3% and 16.0%, respectively).

Reliability of the scales and the frequency of uncontrolled asthma

Reliability testing showed that ACT had high reliability with a Cronbach’s alpha of 0.907. The mean ACT score was 20.49 (SD=5.30), for a range of 6-25. The prevalence of uncontrolled asthma was estimated at 32.7% (95%CI = 25.2-40.8%). On the other hand, AKq showed low reliability (Cronbach’s alpha = 0.558) (Results are not presented in tables).

Socioeconomic and environmental factors of uncontrolled asthma

The frequency of uncontrolled asthma was significantly higher among children with an asthmatic parent (46.7% vs 26.7%; p=0.017) while it was lower among those who are exposed to bakhoor at home (26.9% vs 45.7%, p=0.024), compared to their counterparts respectively. No other socioeconomic or environmental factor showed a statistically significant association with uncontrolled asthma (Table 3).

**Table 3:** Sociodemographic and environmental factors of asthma control n: frequency; %: percentage * Statistically significant result (p<0.05). Test used: t Independent t-test; F Fisher's exact test; otherwise, chi-square test was used.

Factor	Level	Groups		p-value
Child-related factors		Controlled	Uncontrolled	
Age (years), mean (SD)		8.32, (3.71)	9.53, (4.21)	.074^t^
Gender, n (%)	Boy	68 (69.4)	30 (30.6)	
	Girl	33 (63.5)	19 (36.5)	.461
Nationality, n (%)	Saudi	67 (71.3)	27 (28.7)	
	Non-Saudi	34 (60.7)	22 (39.3)	.182
Family factors				
Father’s age (years), mean (SD)		42.72, (8.09)	43.08, (8.74)	.804
Father’s education, n (%)	Low	4 (57.1)	3 (42.9)	
	Intermediate	53 (63.9)	30 (36.1)	
	High	44 (73.3)	16 (26.7)	.413
Mother’s age (years), mean (SD)		36.54, (6.80)	36.47, (5.81)	.947
Mother’s education, n (%)	Low	10 (83.3)	2 (16.7)	
	Intermediate	51 (63.8)	29 (36.3)	
	High	40 (69.0)	18 (31.0)	.380
Household income, n (%)	low	44 (62.0)	27 (38.0)	
	Medium	40 (74.1)	14 (25.9)	
	High	17 (68.0)	8 (32.0)	.359
Number of siblings, n (%)	None	5 (71.4)	2 (28.6)	
	1-2	29 (63.0)	17 (37.0)	
	3-4	39 (65.0)	21 (35.0)	
	5+	28 (75.7)	9 (24.3)	.624
Asthmatic parent, n (%)	Yes	24 (53.3)	21 (46.7)	
	No	77 (73.3)	28 (26.7)	.017*
Environmental factors, n (%)				
Parental smoking	Yes	32 (62.7)	19 (37.3)	
	No	69 (69.7)	30 (30.3)	.390
Other family member smoking	Yes	42 (68.9)	19 (31.1)	
	No	59 (66.3)	30 (33.7)	.743
Presence of plants at home	Yes	7 (63.6)	4 (36.4)	
	No	94 (67.6)	45 (32.4)	.750^F^
Living near industrial zone	Yes	11 (73.3)	4 (26.7)	
	No	90 (66.7)	45 (33.3)	.774^F^
Exposure to pets or farm animals	Yes	8 (66.7)	4 (33.3)	
	No	93 (67.4)	45 (32.6)	1.000^F^
Cat at home during the first year of life	Yes	8 (72.7)	3 (27.3)	
	No	93 (66.9)	46 (33.1)	1.000^F^
Cat at home in the last 12 months	Yes	3 (60.0)	2 (40.0)	
	No	98 (67.6)	47 (32.4)	.662^F^
Bakhoor at home	Yes	76 (73.1)	28 (26.9)	
	No	25 (54.3)	21 (45.7)	.024*
Home hygiene (dust, mold, carpets, etc.)	Yes	31 (60.8)	20 (39.2)	
	No	70 (70.7)	29 (29.3)	.220

Asthma-specific and other health factors of uncontrolled asthma

The prevalence risk of uncontrolled asthma was higher among children who declared having used oral corticosteroids (47.7% vs 26.4%, p=0.011) or antibiotics (41.0% vs 22.4%, p=0.016) during the past 12 months and increased with the number of ICU admissions (p=0.044) (Table 4).

**Table 4:** Asthma-related and other health-related factors associated with asthma control (N=150) n: frequency; %: percentage § diabetes mellitus, hypertension, hyperlipidemia, rheumatological disorder, cardiac, etc. * Statistically significant result (p<0.05)

Factor	level	Groups		p-value
Asthma-specific, n (%)		Controlled	Uncontrolled	
Age at asthma onset	≤2 years	71 (66.4)	36 (33.6)	
	>2 years	30 (69.8)	13 (30.2)	.687
Asthma duration	≤5 years	48 (69.6)	21 (30.4)	
	>5 years	53 (65.4)	28 (34.6)	.591
Oral corticosteroid in past 12 months	Yes	78 (73.6)	28 (26.4)	
	No	23 (52.3)	21 (47.7)	.011*
Antibiotic use in the past 12 months	Yes	49 (59.0)	34 (41.0)	
	No	52 (77.6)	15 (22.4)	.016*
Number of ICU admissions in the past 12 months	None	76 (73.1)	28 (26.9)	
	1	12 (63.2)	7 (36.8)	
	2+	13 (48.1)	14 (51.9)	.044*
Other parameters, n (%)				
History of allergic rhinitis	Yes	35 (60.3)	23 (39.7)	
	No	66 (71.7)	26 (28.3)	.147
History of atopic dermatitis	Yes	15 (60.0)	10 (40.0)	
	No	86 (68.8)	39 (31.2)	.392
Gastroesophageal reflux disease	Yes	10 (62.5)	6 (37.5)	
	No	91 (67.9)	43 (32.1)	.663
Other chronic diseases ^§^	Yes	7 (63.6)	4 (36.4)	
	No	94 (67.6)	45 (32.4)	.786
Breastfeeding	Yes	76 (69.1)	34 30.9)	
	No	25 (62.5)	15 (37.5)	.447

Association of parental asthma knowledge with the level of asthma control among children

Since the AKq showed low reliability, the association of parents’ knowledge with asthma control was analyzed for every knowledge item separately. Results showed a negative association between the level of asthma control and the three knowledge items. That is, parents of children with uncontrolled asthma showed significantly better knowledge in the three AKq items, compared to parents who had children with controlled asthma. The first of the three items was “After a child’s asthma attack, once the coughing is over, use of the inhaler and medications should stop”, answered correctly by 34.7% of parents of children with uncontrolled asthma versus 18.8% of parents of children with controlled asthma (p=0.033). The second item was “if an asthmatic child gets the flu, you should apply the inhalers even if there’s no coughing or wheezing”, answered correctly by 77.6% in the uncontrolled asthma group versus 45.5% in the controlled asthma group (p<0.001). The third item was “if the parents of a child with asthma smoke outside the house, it won’t affect the child”, answered correctly by 55.1% in the uncontrolled asthma group versus 37.6% in the controlled asthma group (p=0.043) (Table 5).

**Table 5:** Association of parental knowledge about asthma with asthma control in children Percentages are calculated on the column categories * Statistically significant results

Knowledge subscale/item	Answer (N)	Level of asthma control, N (%)		p-value
		Controlled (n=101)	Uncontrolled (n=49)	
Myths and beliefs about asthma				
Inhaler use can lead to dependence or addiction	Incorrect	61 (60.4)	31 (63.3)	
	Correct	40 (39.6)	18 (36.7)	.735
Inhalers can have an effect on the heart or damage it	Incorrect	67 (66.3)	37 (75.5)	
	Correct	34 (33.7)	12 (24.5)	.253
It’s not good for children to use the inhaler for too long	Incorrect	77 (76.2)	40 (81.6)	
	Correct	24 (23.8)	9 (18.4)	454
After a child’s asthma attack, once the coughing is over, use of the inhaler and medications should stop	Incorrect	82 (81.2)	32 (65.3)	
	Correct	19 (18.8)	17 (34.7)	.033*
Children with asthma should use asthma medications only when they have symptoms	Incorrect	79 (78.2)	39 (79.6)	
	Correct	22 (21.8)	10 (20.4)	.847
It’s better to use inhalers directly, without a holding chamber, so the medication can go more directly to the lungs	Incorrect	39 (38.6)	24 (49.0)	
	Correct	62 (61.4)	25 (51.0)	.228
When a child has an asthma attack it’s best to go to the emergency room even if symptoms are mild	Incorrect	71 (70.3)	34 (69.4)	
	Correct	30 (29.7)	15 (30.6)	.909
Knowledge about the disease				
The main cause of asthma is airway inflammation	Incorrect	17 (16.8)	11 (22.4)	
	Correct	84 (83.2)	38 (77.6)	.408
Asthma attacks can be prevented if medications are taken even when there are no symptoms between attacks	Incorrect	31 (30.7)	11 (22.4)	
	Correct	70 (69.3)	38 (77.6)	.292
Flu infections are the main causes or triggers of asthma attacks	Incorrect	15 (14.9)	8 (16.3)	
	Correct	86 (85.1)	41 (83.7)	.814
If an asthmatic child gets the flu, you should apply the inhalers even if there’s no coughing or wheezing	Incorrect	55 (54.5)	11 (22.4)	
	Correct	46 (45.5)	38 (77.6)	< .001>
Asthmatic children might have attacks that are severe enough to require hospitalization in an intensive care unit or they might even die from an attack	Incorrect	13 (12.9)	4 (8.2)	
	Correct	88 (87.1)	45 (91.8)	.394
Some medications for asthma don’t work unless they’re administered every day	Incorrect	46 (45.5)	19 (38.8)	
	Correct	55 (54.5)	30 (61.2)	.433
Associated aspects of asthma				
Parents should ask a doctor to tell the school that an asthmatic child shouldn’t exercise or participate in physical education classes	Incorrect	87 (86.1)	39 (79.6)	
	Correct	14 (13.9)	10 (20.4)	.305
Children who have asthma shouldn’t participate in sports that make them run too much	Incorrect	82 (81.2)	36 (73.5)	
	Correct	19 (18.8)	13 (26.5)	.279
It’s best not to smoke or let anyone else smoke near a child who has asthma	Incorrect	19 (18.8)	5 (10.2)	
	Correct	82 (81.2)	44 (89.8)	.177
If the parents of a child with asthma smoke outside the house, it won’t affect the child	Incorrect	63 (62.4)	22 (44.9)	
	Correct	38 (37.6)	27 (55.1)	.043*

Independent factors of uncontrolled asthma

The multivariate regression model showed that exposure to bakhoor at home (OR = 0.41, p=0.044), two or more ICU admissions during the past 12 months (OR = 3.30, p=0.030), and incorrectly answering to the item “if an asthmatic child gets the flu, you should apply the inhalers even if there’s no coughing or wheezing” (OR = 0.22, p=0.001) were the three independent factors of uncontrolled asthma among children. The model explained 31.6% of the outcome variance (R^2^ = 0.316) (Table 6).

**Table 6:** Independent factors associated with uncontrolled asthma (multivariate logistic regression) AKq A4: After a child’s asthma attack, once the coughing is over, use of the inhaler and medications should stop AKq B4: If an asthmatic child gets the flu, you should apply the inhalers even if there’s no coughing or wheezing AKq C4: If the parents of a child with asthma smoke outside the house, it won’t affect the child ^§^ Reference category is "correct answer" * Statistically significant result (p<0.05). Model goodness-of-fit: R^2^ = 0.316

Predictor	Level	OR (95%CI)	p-value
Asthmatic parent	Yes	2.34 (0.97-5.61)	.058
Bakhoor at home	Yes	0.41 (0.17-0.98)	.044*
Oral corticosteroid in past 12 months	Yes	1.02 (0.41-2.52)	.969
Antibiotic use in the past 12 months	Yes	2.03 (0.86-4.76)	.104
Number of ICU admission in the past 12 months	None	(Reference)	.049*
	1	2.52 (0.77-8.22)	.125
	2+	3.30 (1.12-9.71)	.030*
AKq A4	Incorrect answer ^ §^	0.90 (0.35-2.34)	.834
AKq B4	Incorrect answer ^§^	0.22 (0.09-0.54)	.001*
AKq C4	Incorrect answer ^§^	0.44 (0.19-1.01)	.052

## Discussion

Summary of the findings

The present study estimated the frequency of uncontrolled asthma among children and investigated the associated socioeconomic and environmental factors, in addition to parents’ knowledge about asthma. We observed that one-third (32.7%) of children had uncontrolled asthma, which was associated with a few environmental and asthma-specific factors. Additionally, three paradoxical associations were observed between parents' knowledge items and the level of asthma control. Nonetheless, the levels of parents' knowledge showed mixed results with frequent myths and disbeliefs and suboptimal-to-optimal levels of knowledge about the disease. In the multivariate analysis, uncontrolled asthma was independently associated with exposure to bakhoor at home, in a negative relationship (OR=0.41), as well as with multiple ICU admissions (OR = 3.30), and incorrect answer to the item “if an asthmatic child gets the flu, you should apply the inhalers even if there’s no coughing or wheezing” (OR = 0.22).

Frequency of uncontrolled asthma

The observed frequency of uncontrolled asthma (32.7%) is lower than that reported in other local studies among asthmatic children. Bin Saeed et al., in 2014, reported a prevalence of uncontrolled asthma of 59.3% in Riyadh [2]. In 2017, a study from two major hospitals in Riyadh, which included 297 physician-diagnosed asthmatic children aged 3-11 years, found that 60.3% of the participants had uncontrolled asthma symptoms [28]. In 2020, two cross-sectional studies, one from Al Madinah Al Munawarah (n=278) [29] and the other from Riyadh (n=177), revealed that 62.6% and 61.6% of the participants were not adequately controlled, respectively [30]. Although our study showed less concerning figures, these observations from local studies indicate alarming levels of uncontrolled asthma among the pediatric population. This suggests the presence of multiple barriers to achieving the optimal treatment outcome that should be meticulously explored. 

Levels of parental knowledge

We observed that the overall parental knowledge of asthma in Saudi Arabia is insufficient, showing mixed results and frequent myths and misbeliefs. Likewise, Al Otaibi et al. collected responses from 231 parents and guardians about childhood asthma awareness and observed a moderate level of knowledge in the majority of participants (79.6%). Furthermore, misconceptions about asthma medications and adverse behaviors during acute episodes (i.e., administration of homemade or herbal remedies) were identified in almost half of the participants [31]. In another study from Aseer Central Hospital, southwest Saudi Arabia, involving 171 mothers of children with asthma, poor maternal knowledge and adverse behaviors were associated with the female sex of the child, illiterate mother, and younger mother’s age. Although these mothers demonstrated overall adequate practices regarding the management and prevention of asthma attacks, the lowest awareness rates concerned the complications of asthma (10.5% for sleep disorders, 9.9% for respiratory failure, and 9.9% for death) [32].

A lack of adequate parental perceptions about asthma was more noticeable in another Saudi study (n=600), by Abu-Shaheen et al., where 53.5% of the respondents believed that asthma is solely a hereditary disorder, and 60.3% and 32% worried about the adverse effects of inhaled corticosteroids and the risk of drug dependency, respectively [33]. Moreover, Alhammad et al. reported that a high percentage of parents were using asthma medications inadequately, as only 54.2% of the respondents were knowledgeable that salbutamol should be inhaled and only 37.9% knew the correct way to inhale corticosteroids [30]. Internationally, a recent systematic review of eight studies from different countries (three from Saudi Arabia) covering a total of 3,701 parents concluded that the levels of parental awareness and knowledge about asthma care are generally low [34]. This "pandemic" of misinformed parents emphasizes the unmet need for urgent interventions to improve parents’ knowledge, and hence proactive contributions in the day-to-day management and monitoring of asthmatic children.

Factors of uncontrolled asthma

Although several factors were associated with the level of asthma control in unadjusted analysis, one factor has been identified to independently increase the likelihood of uncontrolled asthma and two other factors to independently decrease it. The factor that increased the risk of uncontrolled asthma (by 3.3 fold) was a history of two or more ICU admissions in the past 12 months. The two factors that decreased the likelihood of uncontrolled were paradoxical and were related to exposure to bakhoor at home and inadequate awareness about the necessity of applying inhalers to the asthmatic child in case of flu, even in absence of asthma crisis symptoms; the likelihood of uncontrolled asthma decreased by 57% and 78%, respectively. Inadequately controlled asthma exposes to a greater risk of severe asthma exacerbations that lead to ICU hospitalizations [18]. Furthermore, the present study showed that the more frequent ICU admission, the higher the risk of uncontrolled asthma. This highlights the relevance of using the number of ICU admissions as an indicator for asthma control.

Unexpectedly, there was a negative association between the use of Bakhoor at home and the risk of uncontrolled asthma, which suggests a positive association with asthma control. Such a finding is paradoxical as exposure to bakhoor or incense burning smoke is recognized to have harmful effects on respiratory function, including a decrease in the forced vital capacity (FVC) as shown in a study from Taiwan that included 5010 adolescents [35]. Moreover, according to a survey from Oman, bakhoor was found to be a potent trigger of asthma symptoms, causing wheezing aggravation in asthmatic school-aged children who had more than twice-a-week exposure at home [36]. This paradoxical finding is probably due to parents of children with uncontrolled asthma being more cautious and educated regarding this risk factor.

The literature shows various levels of contribution of the sociodemographic and environmental factors in asthma control among children. For instance, a study from China (n=2,485) showed a positive association between a family history of asthma and poor asthma control [37], which is consistent with our findings in the unadjusted analysis. Likewise, another study from the Netherlands involving 408 children, showed that a family history of asthma increased the odds of uncontrolled asthma by two-fold [38]. One suggested explanation is that children born to asthmatic parents have more severe forms [39], and would consequently be more difficult to manage. In asthmatic children, antibiotics are mainly prescribed for lower respiratory tract infections (potential trigger of disease exacerbation), or as part of the exacerbation’s treatment, although in absence of evidence-based guidelines [40]. Such indications and use explain, in essence, the significant association of antibiotic use with uncontrolled asthma.

Another positive association was found between oral corticosteroid use and uncontrolled asthma in the unadjusted analysis; however, this was not significant in the adjusted analysis. In the pediatric population, oral corticosteroid use is mostly restricted to acute attacks and is prescribed as a short-course regimen given the potential side effects associated with the systemic route [41]. In the context of acute asthma, early administration of systemic corticosteroids has demonstrated its benefits in reducing the risk of emergency visits and hospitalization in asthmatic infants [42], along with lowering the disease relapses [43]. Similar to antibiotics, such indications and uses of corticosteroids may explain the associated higher risk of uncontrolled asthma.

Parents’ knowledge and asthma control

Given that the AKq scale showed low reliability, the present study failed to analyze the association of asthma control with the overall level of parents’ knowledge about asthma. Nevertheless, incorrect answers to three of the AKq items were paradoxically associated with a lesser risk of uncontrolled asthma. This means that children of parents who had lesser knowledge regarding these three items had a higher probability of being adequately controlled. However, only one of these three items, “if an asthmatic child gets the flu, you should apply the inhalers even if there’s no coughing or wheezing” was significant in the adjusted analysis, where an incorrect answer was associated with 78% risk reduction of uncontrolled asthma.

Reportedly, poor parental knowledge about asthma was shown to be an independent factor for uncontrolled asthma [30,44]. For instance, a local study showed that unawareness among parents about their child’s medications’ names increased the odds of uncontrolled asthma by six-fold, whereas parental misconceptions regarding inhaled corticosteroid use increased the odds by two-fold [30]. 

To extend the reflection, there might be a plausible explanation for the paradoxical association of poor knowledge with better asthma control observed in the present study. That is, children who have uncontrolled asthma have more encounters with physicians and healthcare professionals during their frequent exacerbation episodes. Hence, this subgroup of children and parents would receive more education and knowledge transmission about the disease and its management. On the other hand, those with well-controlled asthma are more likely to miss their follow-up appointments, which impacts their level of commitment and knowledge. Intensified and adaptive education and awareness programs should be implemented at all levels of management and follow-up, starting at diagnosis and continuing throughout the follow-up. These programs should target various audiences, address the myths and misbeliefs, and engage both the parents and afflicted children in self and home management.

Study implications

A proactive physician-patient-parent relationship is fundamental for adequate patient and parent education about mitigating the environmental factors of uncontrolled asthma and enhancing adherence and adequate use of the treatments for a better outcome. The better the communication between physicians and parents of asthmatic children, the higher the likelihood of implementing the needed actions by caregivers [45]. Previous studies have demonstrated the effectiveness of family educative programs in optimizing parental awareness about asthma [46,47]. A randomized trial from Iran including 45 school-aged children with their parents tested the effects of a family empowerment program with multiple educational interventions (self-directed educational material, lectures, group interaction and discussions, and screening of educational films) on asthma control. Findings demonstrated a significant improvement in asthma control scores among the intervention group [46]. In another single-center, randomized controlled trial from Tunisia, families that received training sessions exhibited improved levels of asthma symptom control and inhaler use in their children. The program included educational sessions concerning basic information about asthma, the recognition of its symptoms, management of exacerbations episodes, avoidance of triggers, use of asthma medication and inhalation technique, and tools to better communicate with the healthcare provider [47]. Such comprehensive programs should inspire physicians and decision-makers to improve the quality of life and long-term outcomes of asthma, consequently reducing its burden on patients, families, and society. Considering the alarming rates of uncontrolled asthma in Saudi Arabia, an adaptive national strategy should be designed by experts to enable effective and personalized interventions, resources, and objectives for maximized benefit.

Limitations

Our study was mainly limited by the cross-sectional design, the single-center implementation, and the small sample size, which may limit the generalizability of the findings. Another limitation is the use of participant-reported assessments of asthma control and self-assessed knowledge, which may reduce the reliability of the answers and explain the low internal consistency of the knowledge scale, as well as the paradoxical associations of knowledge with the level of asthma control. 

## Conclusions

Uncontrolled asthma concerns one-third of the asthmatic children following at the participating center, representing a relatively less concerning figure than the national data. Several socioeconomic and environmental factors were identified to positively or negatively impact asthma control; these factors should be identified to optimize the identification and management of asthmatic children. In the present study, the level of parental knowledge did not have a significant impact on asthma control. Nevertheless, we noted inadequate levels of knowledge and frequent myths and misbeliefs about asthma, which may impact the adequate home management of children and induce adverse behaviors among parents and caregivers. A proactive physician-patient-parent relationship is fundamental for adequate patient and parent education to manage the environmental factors affecting uncontrolled asthma and improve adherence and proper use of the treatments. An adaptive strategy should be designed to enable effective and personalized interventions, resources, and objectives for maximized benefits.
